# Effects of respiratory mechanics on the capnogram phases: importance of dynamic compliance of the respiratory system

**DOI:** 10.1186/cc11659

**Published:** 2012-10-02

**Authors:** Barna Babik, Zsófia Csorba, Dorottya Czövek, Patrick N Mayr, Gábor Bogáts, Ferenc Peták

**Affiliations:** 1 Department of Anesthesiology and Intensive Therapy, University of Szeged, 6 Semmelweis u., H-6720 Szeged, Hungary; 2 Department of Medical Physics and Informatics, University of Szeged, 9 Korányi fasor, H-6720 Szeged, Hungary; 3 Department of Anesthesiology, German Heart Center, 36 Lazarettstr., D-80636 Munich, Germany; 4 Department of Cardiac Surgery, University of Szeged, 4 Pécsi u., H-6720 Szeged, Hungary

## Abstract

**Introduction:**

The slope of phase III of the capnogram (S_III_) relates to progressive emptying of the alveoli, a ventilation/perfusion mismatch, and ventilation inhomogeneity. S_III _depends not only on the airway geometry, but also on the dynamic respiratory compliance (Crs); this latter effect has not been evaluated. Accordingly, we established the value of S_III _for monitoring airway resistance during mechanical ventilation.

**Methods:**

Sidestream capnography was performed during mechanical ventilation in patients undergoing elective cardiac surgery (*n *= 144). The airway resistance (Raw), total respiratory resistance and Crs displayed by the ventilator, the partial pressure of arterial oxygen (PaO_2_) and S_III _were measured in time domain (S_T-III_) and in a smaller cohort (*n *= 68) by volumetry (S_V-III_) with and without normalization to the average CO_2 _phase III concentration. Measurements were performed at positive end-expiratory pressure (PEEP) levels of 3, 6 and 9 cmH_2_O in patients with healthy lungs (Group HL), and in patients with respiratory symptoms involving low (Group LC), medium (Group MC) or high Crs (Group HC).

**Results:**

S_T-III _and S_V-III _exhibited similar PEEP dependencies and distribution between the protocol groups formed on the basis of Crs. A wide interindividual scatter was observed in the overall Raw-S_T-III _relationship, which was primarily affected by Crs. Decreases in Raw with increasing PEEP were reflected in sharp falls in S_III _in Group HC, and in moderate decreases in S_III _in Group MC, whereas S_T-III _was insensitive to changes in airway caliber in Groups LC and HL.

**Conclusions:**

S_III _assessed in the time domain and by volumetry provide meaningful information about alterations in airway caliber, but only within an individual patient. Although S_T-III _may be of value for bedside monitoring of the airway properties, its sensitivity depends on Crs. Thus, assessment of the capnogram shape should always be coupled with Crs when the airway resistance or oxygenation are evaluated.

## Introduction

Capnography is a noninvasive, continuous, online, dynamic, effort- and cooperation-independent method for bedside monitoring of the exhaled carbon dioxide (CO_2_) concentration. The slope of the third phase of the capnogram (S_III_) is determined physiologically by the continuous CO_2 _excretion from the pulmonary vasculature and the periodic lung ventilation [1,2], and the interactions between the diffusive and convective gas mixing [3-5]. The development of pathophysiological ventilatory and/or perfusion inhomogeneities in time and space leads to lung compartments containing various CO_2 _concentrations, and this may further elevate S_III _[2,6-13].

The sensitivity of S_III _to ventilation/perfusion abnormalities suggested its clinical usefulness for the detection of respiratory abnormalities or the following of ventilatory and/or pharmacological interventions. Numerous studies have demonstrated that the magnitude of S_III _reflects the severity of emphysema or asthma [4,11,13-17], cystic fibrosis and bronchiectasis [8], chronic obstructive pulmonary disease (COPD) [7], chronic bronchitis [9] and acute lung injury [6,10]. Inconsistent associations have been reported in previous attempts to clarify the quantitative relationships between capnographic and lung function indices. Earlier studies reported a strong correlation between the forced expiratory volume in one second (FEV_1_) and S_III _[15], merely a modest association [11,16], or even a lack of correspondence [13]. Furthermore, significant correlations were observed between the total respiratory resistance (Rrs) and S_III _in mechanically ventilated patients, however S_III _had limited clinical applicability to predict Rrs [18]. Thus, in consequence of the complex mechanisms affecting S_III_, its diagnostic and/or monitoring value is far from being clear. The diverse emptying of different lung compartments with various CO_2 _levels is determined not only by the airway geometry, but also by the driving pressure governed by the dynamic respiratory compliance (Crs), including the chest wall and the lung. Despite the obvious importance of respiratory tissue elastance in determining the expiratory flow and the rate of CO_2 _clearance, the role of the respiratory elastic recoil on the capnogram shape has not been examined to date.

The aim of the present prospective consecutive clinical study was to investigate systematically whether the capnogram shape is affected by changes in both airway caliber and the Crs in mechanically ventilated patients. We also set out to clarify the contribution of the altered airway properties and tissue mechanics with increasing positive end-expiratory pressure (PEEP) to the changes in S_III_. To test the hypothesis that both the airway geometry and the Crs reflecting the elastic recoil of the respiratory system affect S_III_, a large cohort of mechanically ventilated patients with normal and diseased lungs was studied.

## Materials and methods

### Patients

One hundred and forty-four patients (93 males, 51 female, 62 ± 9 (mean ± SD) years of age (range 39 to 84 years)) undergoing elective coronary bypass surgery were examined in the supine position before the surgical procedure. The protocol was approved by the Human Research Ethics Committee of Szeged University, Hungary (no. WHO 2788), and the patients gave their informed consent to the study. The patients were premedicated with intramuscular morphine (0.07 mg/kg) and midazolam (0.07 mg/kg) 1 h before the operation. Anesthesia was induced with i.v. midazolam (30 μg/kg), sufentanil (0.4 to 0.5 μg/kg) and propofol (0.3 to 0.5 mg/kg). Muscle paralysis was achieved with an i.v. bolus of rocuronium (0.6 mg/kg). The anesthesia and muscle relaxation were maintained with i.v. infusions of propofol (50 μg/kg/min) and i.v. boluses of rocuronium (0.2 mg/kg every 30 min).

The trachea was intubated with a cuffed endotracheal tube with an internal diameter of 7, 8 or 9 mm, and the patients were ventilated with a Dräger Zeus anesthesia machine (Lübeck, Germany) in volume-controlled mode with descending flow. The ventilator frequency was set to 12 to 14 breaths/min, a tidal volume of 7 ml/kg and a PEEP of 3 cmH_2_O were applied. The fraction of inspired oxygen (FiO_2_) was maintained at 0.5 throughout the entire study period. Arterial blood gas samples were analyzed at least hourly (Radiometer ABLTM 505, Copenhagen, Denmark). The ejection fraction (EF) data were collected from preoperative echocardiography. The body mass index (BMI) of each patient was calculated.

The patients had various cardiac diseases including ischemic heart disease (*n *= 108), a mitral insufficiency (*n *= 21), aortic stenosis (*n *= 38), and other types of cardiac malformation (*n *= 5), such as myxoma, congenital heart disease or aortic aneurysm. The patients exhibited wide variations in their pulmonary status, with some of them having no pulmonary symptoms (that is, no history of lung disease, normal BMI, no pleural effusion, no pulmonary congestion, no smoking history, lack of wheezing periods within the past six months, lack of use of bronchodilator drugs; *n *= 29). Others had lung abnormalities causing restrictive (pulmonary congestion (*n *= 45) or obesity (BMI ≥ 31; *n *= 48)) and/or obstructive changes (emphysema (*n *= 55), asthma (*n *= 14), chronic bronchitis (*n *= 25) or sarcoidosis (*n *= 1)).

To establish whether the elastic properties of the respiratory system affected the capnogram shape, the patients with respiratory symptoms were allocated into three groups, on the basis of their Crs. This Crs was determined 10 min after anesthesia induction and a lung volume homogenization maneuver (that is, lung inflation and maintenance at a transrespiratory pressure of 30 cmH_2_O for 5 s) when stable hemodynamic and ventilatory conditions at PEEP 3 cmH_2_O have been reached (that is, prior to the first capnogram recording). Group LC comprised patients with Crs in the lowest tenth percentile (Crs < 34.5 ml/cmH_2_O; *n *= 15), and Group MC patients with Crs between the tenth and the ninetieth percentile (34.5 < Crs < 69 ml/cmH_2_O; *n *= 85), and Group HC patients with Crs above the ninetieth percentile (Crs > 69 ml/cmH_2_O; *n *= 15). Patients with healthy lungs were regarded as an independent group (Group HL; *n *= 29). The patients were classified based on the Crs measured after a lung volume recruitment maneuver. The characteristics of the patients in each protocol group are summarized in Table 1. The age of the patients did not differ significantly in the different groups (*P *= 0.16).

**Table 1:** Number of patients with different conditions/diagnoses in each protocol group.

	Gender* (m/f)	Obesity* (n/ow/ob)	Pulmonary status E*/A*/CB*/OLD	Cardiac disease CAD/AS/MI/LEF*/OCD
**Group HL (*n *= 29)**	20/9	10/19/-	-/-/-/-	26/3/3/-/1

**Group HC (*n *= 15)**	15/-	8/7/-	14/-/6/-	11/4/-/-/-

**Group MC (*n *= 84)**	56/28	12/28/44	37/10/15/2	61/28/8/10/3

**Group LC (*n *= 15)**	3/12	-/5/10	4/4/4/-	10/3/1/8/1

Obesity categories: n, normal (20 ≤ BMI < 25); ow, overweight (25 ≤ BMI < 30); ob, obese (30 ≤ BMI). Pulmonary status: E, emphysema; A, asthma; CB, chronic bronchitis; OLD, other lung disease. Cardiac disease: CAD, coronary artery disease; AS, aortic stenosis; MI, mitral insufficiency; LEF, low ejection fraction (EF < 50%); OCD, other cardiac disease. Pulmonary and cardiac conditions are based on previous clinical diagnoses. *, *P *< 0.05 between the expected and the observed frequencies in the protocol groups for each variable. Group HC, group of patients with high dynamic respiratory compliance; Group HL, group of patients with healthy lungs; Group LC, group of patients with low dynamic respiratory compliance; Group MC, group of patients with medium dynamic respiratory compliance.

### Measurement of airway and respiratory tissue mechanics

Details of the measurement of the input impedance of the respiratory system (Zrs) were reported previously [19]. Briefly, a T-piece with two collapsible segments was attached to the distal endotracheal tube, with one end connected to the respirator and the other end to a loudspeaker-in-box system. This apparatus allowed switching of the patient from the respirator to the forced oscillatory setup during the recordings. These were performed by generating pseudorandom pressure excitations into the trachea during short (15 s) apneic pauses superimposed into the mechanical ventilation. The forcing signal contained 30 integer-multiple components of the 0.2 Hz fundamental frequency, in the frequency range 0.2 to 6 Hz. Tracheal airflow (V') was measured with a 28 mm ID screen pneumotachograph connected to a differential pressure transducer (ICS model 33NA002D; ICSensors, Milpitas, CA, USA). The airway opening pressure (Pao) was detected with an identical pressure transducer. Zrs was computed from the power spectra of Pao and V', and then ensemble-averaged under each condition. The mean Zrs data were fitted by a well-validated four-parameter model [20] containing a frequency-independent airway resistance (Raw) and inertance (Iaw) and a constant-phase tissue compartment characterized by the coefficients of damping (G) and elastance (H).

### Recording and analyses of the capnogram

Changes in partial CO_2 _pressure in the exhaled gas during mechanical ventilation were measured with a calibrated sidestream capnometer (Ultima™, Datex/Instrumentarium, Helsinki, Finland). Since capnograms are displayed in clinical routine in the time domain, time capnography was applied in each patient to record CO_2 _changes. To minimize the possible drawback of this time domain analyses, we paid attention to involve only the linear part of the CO_2 _trace in the readings of S_III_. Nevertheless, volumetric capnography may allow a better distinction between the phases [2,4,6,7,10,12,14,21] and thus, in a subgroup including the last 68 patients, the flow during mechanical ventilation was simultaneously recorded with the CO_2 _traces by introducing an additional pneumotachograph into the ventilation circuit. This allowed the analyses of volumetric capnograms in 20, 7, 32 and 9 patients in the Groups HL, HC, MC and LC, respectively. The 15 s CO_2 _and respiratory flow traces were imported into commercial signal analysis software (Biopac, Santa Barbara, CA, USA). Linear regression analysis was applied to obtain the slope of the third phase of the expiratory capnogram in the time domain (S_T-III_) and CO_2 _concentration was analyzed as a function of expired volume to obtain the volumetric third phase slope (S_v-III_). These analyses were performed by fitting a line for each expiratory phase in the recordings in the linear phase before the end-expiratory peak [6,11]. Both S_T-III _and S_V-III _were normalized by dividing each slope by the average values of the corresponding CO_2 _concentration in mixed expired gas to obtain normalized time domain (Sn_T-III_) and volumetric (Sn_V-III_) third phase slopes [2,21,22]. Three to four expiratory traces were analyzed in each recording resulting in an ensemble averaging of 10 to 12 values under each condition.

### Measurement protocol

**Figure 1:**
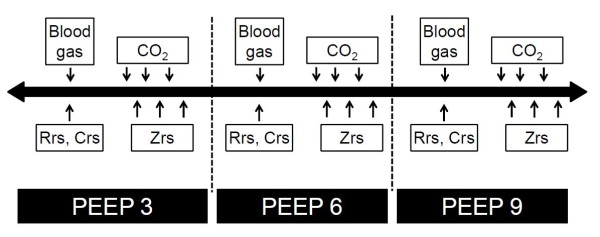
**Timeline of the experimental protocol**. Rrs and Crs, readings from the respirator display; CO_2_, recording of capnogram curves; Zrs, forced oscillatory measurement of respiratory system impedance. CO_2_, carbon dioxide; Crs, dynamic respiratory compliance; Rrs, total respiratory resistance; Zrs, input impedance of the respiratory system.

The scheme of the experimental protocol is outlined in Figure 1. When stable hemodynamic and respiratory mechanical conditions had been reached while PEEP was maintained at 3 cmH_2_O, an arterial blood gas sample was taken, and dynamic compliance (Crs) was recorded from the display of the respirator. The first capnogram trace was then collected followed by recording of the first Zrs data epoch. Two more capnographic and Zrs measurements were then made in alternating sequence at 60 s intervals. PEEP was next elevated to 6 and then 9 cmH_2_O, a 3 min equilibration period being permitted after each step, and the data collection procedure was repeated.

### Statistical analyses

Scatters in measured variables are expressed as SE. The normality of the data was tested with the Kolgomorov-Smirnov test with Lilliefors correction. Two-way repeated measures analysis of variance (ANOVA) including an interaction term was used with the variables PEEP (3, 6 and 9 cmH_2_O) and the group allocation (Groups HL, LC, MC and HC) to establish the effects of lung volume and Crs on the respiratory mechanical, blood gas and capnographic variables. This statistical method was utilized to test the hypothesis that the level of Crs affects the PEEP-dependent changes in the respiratory mechanical and capnogram variables. Multiple linear regression analysis was performed to establish whether the levels of BMI and EF affect Crs. The Holm-Sidak multiple comparison procedure was adopted to compare the variables in the various study groups under different conditions. Chi-square test was used to assess whether there is a significant difference between the expected and the observed frequencies of gender, obesity, pulmonary and cardiac diseases in the protocol groups. The correlation between S_T-III _and S_V-III _were analyzed by Pearson test. The statistical tests were performed with a SigmaPlot statistical software package (Version 12, Systat Software, Inc. Chicago, IL, USA). All reported *P *values are two-sided.

## Results

**Figure 2:**
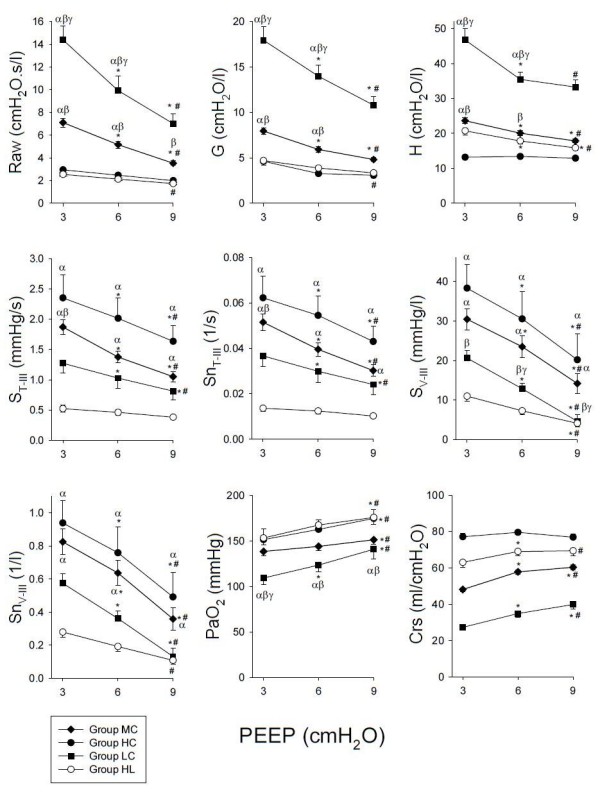
**Forced oscillatory airway (Raw, airway resistance) and respiratory tissue (G, damping and H, elastance) mechanical parameters, the slope of the third phase of the capnogram as expressed in the time domain before (S_T-III_) and after normalization for the mean expired CO2 (Sn_T-III_), or as a function of expired volume before (S_V-III_) and after (Sn_V-III_) normalization, partial pressure of oxygen in the arterial blood (PaO_2_) and dynamic compliance (Crs) displayed by the respirator in patients with healthy lungs (Group HL), and in patients with respiratory symptoms with Crs in the lowest tenth percentile (Group LC), with Crs between the tenth and the ninetieth percentile (Group MC) and with Crs above the ninetieth percentile in (Group HC)**. *, *P *< 0.05 vs. the variable at the previous PEEP level; #, *P *< 0.05 vs. a variable at a PEEP of 3 cmH_2_O. α, *P *< 0.05 vs. Group HL within a PEEP; β, *P *< 0.05 vs. Group HC within a PEEP; γ, *P *< 0.05 vs. Group MC within a PEEP.

The changes in the respiratory mechanical parameters, the partial pressure of arterial oxygen (PaO_2_) and the indices obtained from the capnograms with increasing PEEP in the four groups of patients are depicted in Figure 2. The statistical analyses revealed significant interactions between the group allocation and PEEP, demonstrating that the respiratory compliance exerted significant effects on the responses to PEEP in the forced oscillatory mechanical parameters (*P *< 0.001 for Raw, G and H), for the Crs displayed by the respirator (*P *< 0.001), PaO_2 _(*P *= 0.04), and the capnogram third phase slope variables (*P *< 0.001 for S_T-III _and Sn_T-III_, *P *= 0.003 for S_V-III_, and *P *= 0.002 and Sn_V-III_). Time and volumetric capnogram variables exhibited similar Crs and PEEP dependences, which is also reflected in the significant correlations between S_T-III _and S_V-III _in Groups HL (R = 0.4, *P *= 0.002), HC (R = 0.79, *P *< 0.001), LC (R = 0.45, *P *= 0.02) and MC (R = 0.79, *P *< 0.001).

The greatest Raw, G, H and the lowest PaO_2 _were observed for the patients in Group LC, and these patients generally exhibited the greatest response to PEEP. The patients in Group MC still exhibited elevated Raw, G and H with a more moderate, but still significant response to PEEP changes. The lowest forced oscillatory airway and tissue parameters and the greatest PaO_2 _were obtained in the patients in Groups HL and HC, and their changes with PEEP were generally mild. The capnogram third phase indices were highest in Group HC and somewhat lower in Group MC, with both groups exhibiting marked decreases with increasing PEEP. The variables characterizing the third phase slopes from the capnogram were lowest in the patients in Group HL.

**Figure 3:**
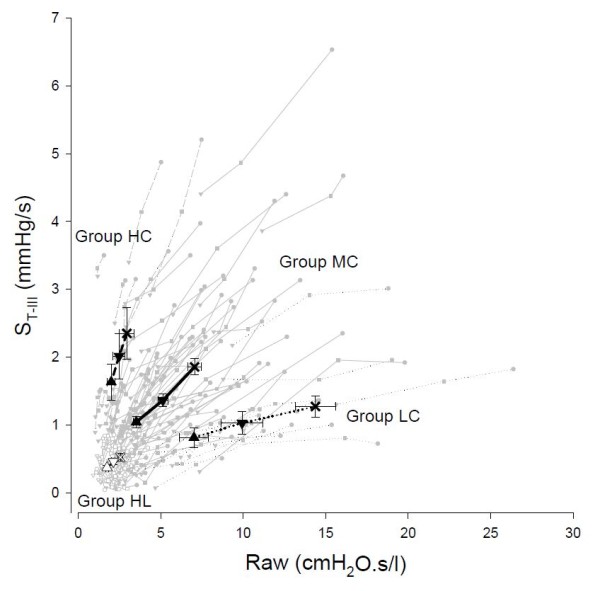
**Relationship between forced oscillatory airway resistance (Raw) and phase III slope of time capnogram (S_T-III_) at PEEP levels of 3 (X), 6 (▼) and 9 cm H_2_O (▲) in patients with healthy lungs (Group HL), and in patients with respiratory symptoms with dynamic respiratory compliance (Crs) in the lowest tenth percentile (Group LC), with Crs between the tenth and ninetieth percentile (Group MC) and with Crs above the ninetieth percentile (Group HC)**. Thin grey lines denote individual patients; thick black lines with symbols show group mean and SE values.

Figure 3 depicts the relationship of Raw and S_T-III _in the individual patients and the group means for the four protocol groups following the increases of PEEP. In all patients, Raw and S_T-III _underwent concomitant monotonous decreases with increasing PEEP, but marked differences were observed between the protocol groups in the relationships of these parameters. The marked decreases in the high initial Raw values were associated with substantially smaller drops in S_III _in the patients in Group LC, whereas the PEEP-induced decreases in S_III _were more pronounced than those in Raw in the patients in Group HC. The patients in Group MC exhibited an intermediate Raw-S_T-III _relationship. This trend of association was observed in the patients in Group HL at markedly lower levels of Raw and S_III_.

**Figure 4:**
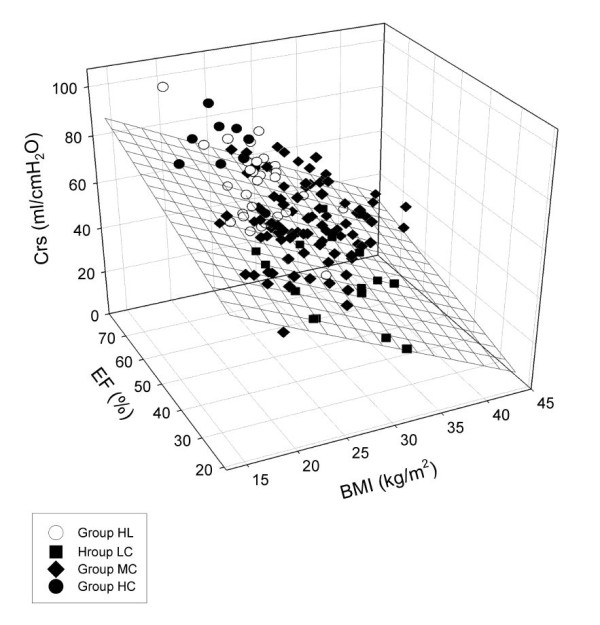
**Effects of body mass index (BMI) and ejection fraction (EF) on dynamic respiratory compliance (Crs)**. The best fit plane is demonstrated with a mesh surface.

To examine the possible roles of obesity and lung congestion in the increased level of Crs, the effects of BMI and EF were considered (Figure 4). The patients in Group LC had significantly higher BMI (*P *< 0.001) and/or lower EF (*P *< 0.001) than those in Groups HL or HC, indicating that the low Crs was a consequence of restrictive changes resulting from obesity and/or heart failure leading to pulmonary congestion (multiple linear regression coefficient of R = 0.58). The important effects of BMI and EF on the group allocation was confirmed by the presence of a significant correlation (R = 0.53, *P *= 0.005 and *P *< 0.0001 for EF and BMI, respectively).

## Discussion

The changes in airway and respiratory tissue mechanics were compared with capnogram third phase indices in a relatively large cohort of patients to clarify its monitoring value in mechanically ventilated patients. Capnogram third phase slopes expressed in the time domain or as a function of expired volume exhibited similar PEEP dependencies and distribution between the protocol groups. Detailed analysis of the time capnogram revealed a strong association between Raw and S_T-III _when the respiratory mechanics was altered by increasing PEEP, which was significantly affected by the degree of expiratory driving pressure of the respiratory system. Accordingly, grouping of the patients based on their Crs revealed that i) the decrease in Raw with increasing PEEP was reflected in a sharp decrease in S_T-III _in patients with low Crs, ii) the increase in airway diameter with increasing PEEP was still reflected in a pronounced decrease in S_T-III _in patients with intermediate Crs, and iii) S_T-III _was insensitive to changes in airway caliber when the Crs was high.

The extent of emptying of lung compartments containing various CO_2 _concentrations during mechanical ventilation and the shape of the resulting capnogram are determined by the airway geometry (that is, the resistance) and the elastic recoil of the respiratory tissues (that is, the driving pressure). Whereas the former has been investigated extensively [4,6,12,13,15], the importance of the latter factor remained unknown. While the time capnogram is most commonly used in clinical practice during mechanical ventilation, distinction of the second and third phases is not always trivial from the time domain analyses and this approach also excludes the consideration of the absolute concentration of CO_2 _in the expired gas. Therefore, we also performed volumetric capnography in a subgroup of patients and normalized the capnogram third phase slope. The similar picture of these different slopes and the significant correlation between them demonstrates that the S_T-III _used in clinical practice can provide relevant information about lung emptying. With the aim of acquiring a general picture, the bedside Crs was used in the present study to group the patients. A strong correlation was earlier demonstrated between the respiratory elastance derived from H and the Crs [23], which justifies the choice of Crs as an appropriate indicator of the respiratory recoil.

We formed four groups with regard to the clinical symptoms (healthy lungs) and the Crs values (diseased lungs with low, medium or high Crs). As expected, the variables reflecting resistive behavior were lowest in the Group HL and they had intermediate H and Crs, permitting fast emptying of the relatively homogeneous lungs, which then results in low capnogram third phase slope, and good PaO_2 _(Figure 2). The increase of PEEP to 6 cmH_2_O caused no further improvement. The slight, but significant decreases in Raw and H and increases in Crs and PaO_2 _at PEEP 9 cmH_2_O may be a consequence of lung recruitment. The lack of decrease in the third phase slope indicates that this opening was relatively uniform in the lung periphery.

The patients in Group HC exhibited similar resistive properties to those in Group HL. However, the high Crs and low H may be a consequence of the loss of elastic recoil in the respiratory tissues, most probably due to emphysematous destruction, which was present in the vast majority of the patients in this group (Table 1). The presence of ventilation heterogeneities is also apparent from the highest third phase slope indices. These can be explained by the existence of peripheral lung units with different small airway calibers and local time constants, resulting in a heterogeneous working lung. This structure leads to gas compartments containing variable CO_2 _concentrations and also results in different local expiratory flows [11]. These phenomena contribute to the sequential emptying of the lung periphery in time, which then increases the time domain and volumetric S_III _values [3-5,10,24]. Since Raw reflects mainly the flow resistance of the central conducting airways [19,23,25], this parameter is not able to detect such alterations in the presence of emphysematous changes [26]. This pathology diminishes predominantly the expiratory flow, while the filling of the lung during mechanical ventilation may remain unaffected or even increased, explaining why PaO_2 _was close to normal. The elevation of PEEP in Group HC decreased G, which is a prerequisite of decreased ventilation heterogeneities with alveolar recruitment [27,28], reflecting in lower capnogram third phase slopes, H, higher Crs and better PaO_2 _[12].

The worst respiratory mechanics and the lowest PaO_2 _were observed in the patients in Group LC. Despite this striking difference, S_III _expressed in time or by volumetry did not differ significantly from those observed in Group HL. This leads to the important observation that even hypoxemia may be associated with a medium level of capnogram third phase slopes, which corresponds to the limited value of capnometry in the assessment of adequate blood oxygenation in this pathology [29,30]. These results can most probably be attributed to the presence of lung regions that remain closed throughout the entire ventilator cycle, leading to some relatively open and fairly uniform working lung units and other, permanently closed, atelectatic lung units. In other words, the closing capacity in these lungs is expected to be higher than the sum of the functional residual capacity and the tidal volume. The persistent lung volume loss with subsequent decrease in the overall airway cross-sectional area is probably reflected in the substantially elevated Raw. Since PEEP elevation may be able to reopen these atelectases, the involvement of these phenomena is substantiated by the most pronounced decreases in the mechanical parameters with increasing PEEP resulting in lower S_III _and elevated PaO_2_, which corresponds to earlier results on similar stiff lungs [10]. Our results confirm previous clinical observations [9,12,25] that this pathophysiology can be triggered by obesity and/or lung congestion arising from a poor EF (Table 1). Taking into account the individual and the combined effects of BMI and EF revealed that low EF or high BMI themselves may be responsible for the compromised Crs. However, the combination of such pathologies exerts additional detrimental effects that lower Crs even more dramatically (Figure 4).

Group MC comprised patients with pulmonary pathologies with an intermediate Crs, a cohort that can be characterized by somewhat elevated airway and respiratory tissue parameters, and ventilation heterogeneities reflected in abnormally high capnography slope characteristics at a PEEP of 3 cmH_2_O. This variable and the intermediate response to PEEP can be explained by concomitant presence of phenomena existing in Groups HC and LC, that is, combined effects of expiratory flow limitation and persistent atelectases.

The overall Raw-S_T-III _relationship was not strong enough to predict the value of Raw from S_T-III _(Figure 3), in agreement with previous findings [6,18]. However, the changes in S_T-III _within an individual patient were appropriate for an assessment or revealing trends of the altered Raw. It should be noted that the Raw-S_T-III _relationship within a patient was highly dependent on the elastic recoil of the respiratory system. In the case of a small Crs, a minor change in S_T-III _may reflect major alterations in airway patency. In contrast, large alterations in S_T-III _may still be associated with small variations in Raw if Crs is high. This finding may explain the controversy in the literature concerning the presence or absence of a correlation between lung function parameters and capnogram indices [11,13,15,16].

The limitations of this study relate to the possible presence of complex cardiopulmonary pathologies within a given patient. The coexistence of opposing factors such as emphysematous changes and a poor left ventricular function precludes identification of the individual effects of pulmonary diseases on the course of the capnogram. An additional aspect is that the surgery did not allow a more time-consuming randomization of the PEEP levels. However, care was taken to provide sufficient time following a change in conditions so that equilibrium was reached, similarly to that allowed following PEEP changes in severe COPD patients [27]. Another methodological limitation is related to the complex effects of PEEP including modification of the lung perfusion [2], increased functional residual capacity [21,31], which may all bias the changes in S_III _and/or the mechanical parameters. However, our results are consistent even on PEEP 3 cmH_2_O alone and the PEEP changes can be considered as reinforcement of the results and mechanisms that existed already at the lower PEEP. Auto-PEEP may be another important factor imposing a potential error with this bias being the most apparent in the patients with high Raw (that is, Group LC) or low driving pressure and compromised emptying of emphysematous destructed alveoli (that is, Group HC). Excluding the auto-PEEP would even enhance the Raw dependence with PEEP, since Raw would theoretically be even higher if auto-PEEP would have been ruled out. Another important feature of the present study is the use of Crs to separate the study groups. Since this parameter incorporates lung and chest wall properties, a separate assessment of which of these compartments are responsible for the altered elastic recoil of the respiratory system is not possible.

## Conclusions

In summary, measurement of the respiratory mechanics and analysis of the capnogram slope demonstrated that changes in S_III _expressed in time or by volumetry provide useful information concerning alterations in airway caliber, but only within an individual patient. The assessment of S_T-III _during mechanical ventilation may be of value for bedside monitoring of the airway resistance, but its sensitivity depends on the elastic recoil of the respiratory system. S_T-III _exhibits high sensitivity to detect changes in the airway resistance in case of high Crs, when the lung emptying is governed primarily by the small airway and alveolar geometry. In cases of stiff respiratory tissues, however, S_T-III _displays low sensitivity in indicating changes in airway caliber, when the lung emptying is determined by the high elastic recoil and depends less on the small airway geometry. The relatively low S_T-III _may coincide with the compromised PaO_2 _in these patients, which suggests that a low S_T-III _does not predict appropriate oxygenation. Thus, the shape of the capnogram should always be evaluated bedside in conjunction with Crs. The joint assessment of the capnogram and the respiratory mechanics is of particular importance in clinical situations when patients with a high BMI and/or a compromised left ventricular function are anesthetized and ventilated.

## Key messages

• The phase III slope of the capnogram evaluated in the time domain or by volumetry exhibits similar PEEP dependencies and distribution between the protocol groups formed on the basis of Crs.

• Crs significantly affects the sensitivity of the phase III slope of the capnogram in the time domain (S_T-III_) and by volumetry to airway dimensions.

• S_T-III _detects changes in the airway resistance sensitively in cases of high Crs.

• S_T-III _displays low sensitivity in cases of stiff respiratory tissues, in indicating changes in airway caliber.

• In conclusion, assessment of the capnogram shape should always be coupled with Crs when the airway resistance or oxygenation are evaluated.

## Abbreviations

BMI: body mass index; CO_2_: carbon dioxide; COPD: chronic obstructive pulmonary disease; Crs: dynamic respiratory compliance; EF: ejection fraction; FiO_2_: fraction of inspired oxygen; G: respiratory tissue damping; Group HC: group of patients with high dynamic respiratory compliance; Group HL: group of patients with healthy lungs; Group LC: group of patients with low dynamic respiratory compliance; Group MC: group of patients with medium dynamic respiratory compliance; H: respiratory tissue elastance; Iaw: airway inertance; Pao: airway opening pressure; PaO_2_: partial pressure of arterial oxygen; PEEP: positive end-expiratory pressure; Raw: airway resistance; Rrs: total respiratory resistance; S_III_: slope of phase III of the capnogram; Sn_T-III_: normalized third phase slope of the expiratory capnogram in the time domain; Sn_V-III_: normalized volumetric third phase slope of the expiratory capnogram; S_T-III_: third phase slope of the expiratory capnogram in the time domain; S_v-III_: volumetric third phase slope of the expiratory capnogram; V': tracheal airflow; Zrs: input impedance of the respiratory system.

## Competing interests

The authors declare they have no competing interests in relation to this manuscript.

## Authors' contributions

BB conducted the design of the study and had a major role in data collection and drafting the manuscript. CsZs and PNM helped with the data collection and analyses. BG performed the surgical preparation and helped with the measurements. CD participated in the study design, data collection and helped with processing the data. FP supervised the data collection and analyses, contributed to the development of the study design and in the manuscript preparation. All authors read and approved the final manuscript.
